# Supplementary material for: The variable presence of Leptospira in the environment; an epidemiological explanation based on serial analysis of water samples
Source: PLoS One. 2022 Feb 15;17(2):e0263719. doi: 10.1371/journal.pone.0263719 (PMC8846531; doi:10.1371/journal.pone.0263719)
Supplement: Attachment — Submitted filename: Response to reviewers.docx [file pone.0263719.s001.docx]

2021.11.04

The Editor,

PLoS ONE.

Dear Editor,

Submitting the replies for the comments by the reviewers of the manuscript titled “The variable presence of *Leptospira* in the environment; An epidemiological explanation based on serial analysis of water samples”

Thank you very much for the extensive review done with regards to the manuscript submitted by us. We highly appreciate the efforts and agree with all the comments. Herewith, we are submitting the replies to the comments and one to one explanation. We are submitting the track changes together with cleaned version. In addition, we did few other corrections for better clarity.

As suggested by the editors, I am including the corrected funding statement. The corrected funding statement is;

“SA: JW and IS are partially supported by US Public Service Grant U19AI115658. The sample analysis was funded through the Faculty of Medicine and Allied Sciences annual publication award received by the first and last authors. All the other costs are self-funded.”

We would be grateful, if you could take necessary actions, for these corrections and accept the paper.

We would like to do further corrections where necessary.

Point by point explanation is mentioned below.

Thank you

Prof. Suneth Agampodi

| **Journal requirements** | | |
| --- | --- | --- |
| **No** | **Comment** | **Reply** |
| 1 | Please ensure that your manuscript meets PLOS ONE's style requirements, including those for file naming. | We re-edited according to the PLOS ONE guidelines. However, we would like to change if anything is missing in the revised article. |
| 2 | "Funding Information and Financial Disclosure sections do not match: | Thank you for showing the mistake.  Funding information mentioned in the manuscript is correct.  As you suggested, we have removed funding section from the manuscript.  The correct statement is,  “SA: JW and IS are partially supported by US Public Service Grant U19AI115658. The sample analysis was funded through the Faculty of Medicine and Allied Sciences annual publication award received by the first and last authors. All the other costs are self-funded.”  This means, during the period of study SA, JW and IS were partially supported by this fund but these funds were not utilized for this study. So we removed funding source of “US Public Service Grant U19AI115658” from the online submission system.  All the analysis were done using the Annual publication award received from Rajarata University of Sri Lanka. So we kept that funding source in the online system.  Please let me know, if further corrections are needed.  We have included this in the cover letter as you have suggested. |
| 3 | We note that you have provided funding information that is not currently declared in your Funding Statement. However, funding information should not appear in the Acknowledgments section or other areas of your manuscript. We will only publish funding information present in the Funding Statement section of the online submission form.  Please remove any funding-related text from the manuscript and let us know how you would like to update your Funding Statement. Currently, your Funding Statement reads as follows:   “SA: JW and IS are partially supported by US Public Service Grant U19AI115658. The sample analysis was funded through the Faculty of Medicine and Allied Sciences annual publication award received by the first and last authors. All the other costs are self-funded. The funders had no role in study design, data collection and analysis, decision to publish, or preparation of the manuscript.” |  |
| 4 | Please note that in order to use the direct billing option the corresponding author must be affiliated with the chosen institute. Please either amend your manuscript to change the affiliation or corresponding author, or email us at plosone@plos.org with a request to remove this option. | The manuscript is amended with the institution of corresponding author.  **^4^Section of Infectious Diseases, Department of Internal Medicine, School of Medicine, Yale University** |
| 5 | We note that Figures 2,3 and 4 in your submission contain [map/satellite] images which may be copyrighted……………………… | Thank you very much for the comment. We understand the concern. Please see the comments for each of the figure.  **Figure – 2** – This is not a copyrighted material. We hand created this map using photoshop by observing an original map.  We have published a previous version of this map, in the PLOS ONE in the following publication. Warnasekara J and Agampodi S are authors of both papers.  Warnasekara J, Agampodi S, Abeynayake R R. Time series models for prediction of leptospirosis in different climate zones in Sri Lanka. *PLoS One*. 2021;16(5):e0248032. Published 2021 May 14. doi:10.1371/journal.pone.0248032  The graphic designer of the both papers is same. (Dr. Gihan Rathnayaka-Acknowledgement section). Figure 2 is an edited version of previous figure.  Therefore, we confirm that there will not be any copyright issue in the figure 2.  **Figure – 3** – This is not a copyrighted material. We created this using photoshop.  The graphic design was done by Dr. Gihan Rathnayaka. (acknowledgement section)  Therefore, we confirm that there will not be any copyright issue in the figure 3.  **Figure – 4** – This is a copyrighted material. We agree with that. We have included a newly created map drawn using photoshop.  Therefore, we confirm that there will not be any copyright issue in the figure 4 with the new addition |
| **Reviewer 1** | | |
| 1 | In this epidemiologic study the authors collected soil and water samples from several regions (wet, dry and semi-dry) of Sri Lanka based on probable exposure history of confirmed leptospirosis patients and tested it by PCR. There are few leptospirosis epidemiologic studies from Sri Lanka. From the wet area 2/12 water sites were positive. From the dry area 1 well in areas of high human contact were positive. A specific positive site in the dry area was chosen to do serial sample testing from 10 wells surrounding a positive well (nr 9) and they found that at different times 4 to 6 of the 10 wells in that site were positive for Leptospira. The work has merit but as described in this paper is very difficult to follow and the abstract does not capture the essence or importance of the work done. The description of the method to collect the samples including a better flow chart, figures, tables and writing to need to be comprehensively revised. | Thank you very much for this comment. We understand that a new reader can find it difficult to capture the whole idea within the text and the clarity can be improved by doing the suggested changes. Following changes were made in the manuscript.   - Figure 1 is changed into a more comprehensive one. Please see the figure 1. - We did several changes in the abstract and in the main manuscript to minimize confusions. - Track changes were shown. - In addition, In the main text, 3 sub sections were made to separate the three components of the study. |
| 2 | If the primers detect nonpathogenic Leptospira species, what is the rationale of detecting virulent species in the water and soil samples? | Thank you for the question. I am not sure ‘to what part/sentence that this statement is referring to. We agree that there are few confusing statements are there in the manuscript.  General explanation is;  General interest is to detect pathogenic *Leptospira.* So, we used pathogenic specific primer for PCR of water. We mentioned that in the methods section under PCR description.  However, in, next generation sequencing of soil samples, detects the organisms up to the level of Genus but not to the level of species. All pathogenic and non-pathogenic species were classified under genus *Leptospira.* Therefore, we can’t differentiate pathogenic and non-pathogenic *Leptospira* in soil samples from the results of this manuscript.  We changed the title of table one as follows.  ***Presence of pathogenic Leptospira in environmental surface water samples and Genus Leptospira in soil samples from dry, wet and intermediate zones in Sri Lanka.***  Also, following change was made in the results section.  *The highest relative abundance (105.6) was reported from one of the samples of site Rathnapura 1 of the wet zone (Figure 2). Thus, these soil samples can comprise both pathogenic and non-pathogenic species, and non-pathogenic species are ubiquitous.*  In addition, following changes were made in the discussion.  *However, the genus Leptospira detected in soil samples includes both pathogenic and non-pathogenic species, although the primer used for PCR of water samples specifically targeted pathogenic Leptospira.*  *Nevertheless, the observed difference may have been due to the different techniques that we performed for soil and water, which is a major consideration and limitation when interpreting the findings.* |
| 3 | Very confusing descriptions of all data. Throughout the paper (including abstract) avoid using Components 1, 2, 3 and just define the sites as 1-X nr of water and soil samples from Wet area, 2- X nr of Wells in Dry Area, 3 – 10 wells surrounding a positive well in dry area. | Thank you very much. We understand your concern.  We minimized using the term component in the manuscript. We provided brief description together with the term component in required sentences for better clarity. We did this throughout the manuscript. The track changes are shown in the manuscript. However, we kept the word component in some places where there is explanation for the component.  In addition, we changed the figure 1.  Also, we revised the whole manuscript again and did several changes to improve the clarity. All the track changes are shown.  In addition, In the main text, 3 sub sections were made to separate the three components of the study for better clarity. |
| 4 | Down the claims of positive Leptospiral transmission routes or correlations with disease hotspots since no clinical data is shown. | Thank you very much and we agree with the statement.  The objective of this paper is not to correlate the findings of this data with clinical data.  To explain further, we changed the following sentence in the discussion. This will avoid mis interpretation.  *Although, we are not intended to compare the results of this study with the clinical data, the finding that two sites in the wet zone were positive while none of the sites in the dry and intermediate zones were positive for Leptospira is compatible with the reported incidence of leptospirosis, as the wet zone reports nearly two times the number of cases compared to that in the dry zone.* |
| 5 | It is difficult to design any prophylactic measures based on an epidemiological study. Reservoir based studies or other specific animal study can therefore open such avenues to search for a better alternatives than available Doxycycline. | Thank you. We agree. We change the following sentence in the discussion to avoid mis interpretation.  *Epidemiological studies alone cannot decide specific prophylactic measures as a replacement for doxycycline. Therefore, novel studies on the prevention of leptospirosis via changes in soil abundance, biological prevention, prevention through environmental toxins, avoiding entry to the body and changing the host response by immune modulation can be explored as new avenues of leptospirosis prevention research in the future.* |
| Reviewer 2 | | |
| 1 | This is a nice manuscript describing findings of Leptospira in the environment, adding to our knowledge of transmission of Leptospira in the environment. | Thank you very much for the compliment. |
| 2 | The authors use "random" to describe Leptospira in the environment, but I'm not sure that's the correct term. Maybe transient? Or variable? Maybe conditions have to be ideal to reach levels above the limit of detection, and with clear correlations of lepto cases in humans and rainy seasons, and as shown in this study, proximity to a lake where feral animals are prevalent, I'm not sure "random" should be used. | Thank you for the important insight. We also think the random is not suitable.  The term transient also giving some conclusiveness and therefore it is also not suitable.  Therefore, we thought to use the term variable instead of random. We changed the manuscript with track changes. We also changed the figure 6.  We also changes the title of the manuscript. |
| 3 | Can the authors justify why they tossed the first centrifuged water pellet? It's mentioned in limitations that they may have lost some lepto with the pellet. Also, a DNA blood mini kit was used for environmental water - can the authors justify the extraction kit use, or were they able to determine whether or not PCR inhibitors were present? | Thank you for the important question. We use the first step to remove debris from the sample. Also, this is helpful to minimize the concentration of inhibitors present in the water. As centrifugation is for short duration, the organism missing is minimal. To avoid confusion, we changed the following sentence.  *Therefore, centrifugation was conducted in two steps. First, samples were centrifuged at 3000 rpm for 5 minutes to allow the debris to be deposited in the bottom of the tube.*  **For the DNA mini kit issue; please see the following explanation.**   - We also had the similar question before starting the research. However, due to resource limitation and unexpected delays of the supplies, we searched for a method to conduct a study using the available resources. - We found the paper published by Julie Vein et al. who performed similar DNA extraction kit and received successful results. - Also, we found that the paper published by Irina et al. compared three DNA kits including the our one. Although, they received significantly higher results by the water specific kits, DNA blood mini kit also gave positive results with adequate mean concentration. - As a result, we went ahead with the available resources based on the scientific evidence. - For this issue, we added a sentence as follows in methodology.   *This extraction kit is validated for environmental water by Julie Vein et al.*   - Also, we added the following sentences to the limitation section citing the Irina et al.   *Also, the DNA extraction kit we used was a tissue and blood kit. This was optimized for environmental water samples by Julie Vein et al. (27) However, evidence suggest that the use of water specific DNA extraction kit enhance the DNA yield.(41)*   - The references for the statement are;   Vein J, Perrin A, Berny PJ, Benoit E, Leblond A, Kodjo A. Adaptation of a real-time PCR method for the detection and quantification of pathogenic leptospires in environmental water. Canadian Journal of Microbiology. 2012 Jul;58(7):828–35.  Riediger IN, Hoffmaster AR, Casanovas-Massana A, Biondo AW, Ko AI, Stoddard RA. An Optimized Method for Quantification of Pathogenic Leptospira in Environmental Water Samples. Stevenson B, editor. PLOS ONE. 2016 Aug 3;11(8):e0160523.  **With regards to the presence of Inhibitors, please see the following explanation.**   - We have not manually checked for PCR inhibitors. - However, the manual of DNA extraction kit clearly mention the following.   The purified DNA is free of protein, nucleases, and other contaminants or inhibitors. |
| 4 | Can the authors describe in better detail the definition of PCR positivity? It's not clear to me how many "replicates" were done, and whether the replicates were in the PCR reaction or replicate samples. | We understand the confusion. We changed the sentences in the methodology section as follows.  *As mentioned above samples from same site were pooled and pooled sample was used for the DNA extraction. Three replicates from each pooled sample were included in PCR analysis. A positive curve with melting temperature was considered a positive replicate. If only one replicate was positive, the sample was considered positive. If two or more replicates were positive, the site was considered strongly positive.* |
| 5 | What is the difference between an "abundant paddy field" and a paddy field (line 183)? More vegetation? Larger? | Thank you very much. This is a spelling mistake.  This should be replaced by the term abandoned. We edited the manuscript. |
| 6 | It might be helpful in the results section to clarify that the soil sequencing detected both pathogenic and non-pathogenic Leptospira, and that non-pathogenic Leptospira are considered ubiquitous. | Thank you or the suggestion. We added a new sentence as follows.  *Both pathogenic and non-pathogenic species can be comprised in these soil samples and non-pathogenic species are ubiquitous.* |
