# Supplementary material for: The variable presence of Leptospira in the environment; an epidemiological explanation based on serial analysis of water samples
Source: PLoS One. 2022 Feb 15;17(2):e0263719. doi: 10.1371/journal.pone.0263719 (PMC8846531; doi:10.1371/journal.pone.0263719)
Supplement: Attachment — Submitted filename: Reply to reviewers plos one soil.docx [file pone.0263719.s002.docx]

**Reply to reviewers**

| **Comment** | **Reply** |
| --- | --- |
| **Journal Requirements** | |
| Please review your reference list to ensure that it is complete and correct. | Done. |
| If you have cited papers that have been retracted, please include the rationale for doing so in the manuscript text, or remove these references and replace them with relevant current references. Any changes to the reference list should be mentioned in the rebuttal letter that accompanies your revised manuscript. | No retracted papers are cited. |
| **Reviewer 1** | |
| Abstract Line 23: define the classic triad | Thank you very much for the comment. We included the epidemiological triad within brackets in the given place. |
| Methods: Line 105 and others: Purposive? What does this word mean? | Thank you very much for the comment. We understand the concern. We used the term ‘purposive’ to indicate the non-probability sampling technique. However, the term purposive is more general.  The term purposive was used in two places to explain the sample selection technique of two components of the study.  Actually, we selected the sites for the first component based on the probable sites of contamination by leptospirosis patients. These were during our clinical studies, investigators past experiences or the experiences of the physicians.  We selected the sites for the second component considering the possibility of daily exposure by the humans.  For, better clarity, we changed the sentences as follows.  *“For the water samples, water sources were selected purposefully based on the probable sites of contamination of diagnosed leptospirosis patients. Sampling was conducted at 12 sites representing all three climatic zones: dry, wet, and intermediate.”*  *“For the second component, water collection sites were selected purposefully considering the possibility of daily human contacts.”* |
| Discussion Line 279: How much is “a satisfactory concentration of Leptospira needed in the environment”. | I humbly thank you for this comment. This is something we can’t give an exact answer and this depends on several factors as shown in the last figure.  However, we needed to explain that the higher concentration due to recent animal contamination could be a factor associated with this.  Further studies on satisfactory concentration could be an interesting research area to explore.  We added the following sentence at the end of the paragraph.  *“Studies on required minimal leptospira concentration in the environment could be new research area to be explored.”* |
| Line 309: This concept of intermittent positivity versus repetitive contamination should be mentioned in the abstract. | Thank you very much for the nice suggestion. We added the following sentence to the abstract.  *“This intermittent nature of positivity could be explained by the repetitive contamination by animal urine.”* |
| **Reviewer 2** | |
| Thank you, the revision provided clarity and I think the manuscript provides valuable information about Leptospira in the environment in an endemic area. I have only one small comment, to de-capitalize "leptospirosis" in the middle of a sentence. | Thank you very much. We did the changes in the manuscript. |
